# Responses of the marine diatom *Thalassiosira pseudonana* to changes in CO_2_ concentration: a proteomic approach

**DOI:** 10.1038/srep42333

**Published:** 2017-02-09

**Authors:** Romain Clement, Sabrina Lignon, Pascal Mansuelle, Erik Jensen, Matthieu Pophillat, Regine Lebrun, Yann Denis, Carine Puppo, Stephen C. Maberly, Brigitte Gontero

**Affiliations:** 1 Aix Marseille Univ, CNRS, BIP, IMM, 31 Chemin J. Aiguier, B.P. 71, 13 402 Marseille, Cedex 20, France; 2 Plate-forme Protéomique, Marseille Protéomique (MaP) IBiSA labelled, Institut de Microbiologie de la Méditerranée, FR 3479, CNRS, 31 Chemin Joseph Aiguier, B.P. 71, 13402 Marseille Cedex 20, France; 3 Aix Marseille Univ, CNRS, INSERM, Institut Paoli-Calmettes, CRCM, Marseille Protéomique, Marseille, France; 4 Plate-forme Transcriptomique, Institut de Microbiologie de la Méditerranée, FR 3479, CNRS, 31 Chemin Joseph Aiguier, B.P. 71, 13402 Marseille Cedex 20, France; 5 Lake Ecosystems Group, Centre for Ecology & Hydrology, Lancaster Environment Centre, Library Avenue, Bailrigg, Lancaster LA1 4AP UK

## Abstract

The concentration of CO_2_ in many aquatic systems is variable, often lower than the K_M_ of the primary carboxylating enzyme Rubisco, and in order to photosynthesize efficiently, many algae operate a facultative CO_2_ concentrating mechanism (CCM). Here we measured the responses of a marine diatom, *Thalassiosira pseudonana*, to high and low concentrations of CO_2_ at the level of transcripts, proteins and enzyme activity. Low CO_2_ caused many metabolic pathways to be remodeled. Carbon acquisition enzymes, primarily carbonic anhydrase, stress, degradation and signaling proteins were more abundant while proteins associated with nitrogen metabolism, energy production and chaperones were less abundant. A protein with similarities to the Ca^2+^/ calmodulin dependent protein kinase II_association domain, having a chloroplast targeting sequence, was only present at low CO_2_. This protein might be a specific response to CO_2_ limitation since a previous study showed that other stresses caused its reduction. The protein sequence was found in other marine diatoms and may play an important role in their response to low CO_2_ concentration.

Diatoms (Bacillariophyceae) are one of the major primary producers in the ocean responsible for about one fifth of the photosynthesis on Earth and are present in virtually all aquatic habitats^1^. Diatoms are believed to be derived from a secondary endosymbiotic process, between a heterotrophic eukaryotic cell, a red alga and possibly a green alga^1^,^2^,^3^. As a consequence of their evolutionary history, diatoms have unique biochemical pathways, such as the presence of a urea cycle, that is absent in other photosynthetic organisms^4^ and different regulatory mechanisms^5^,^6^. These novel metabolic capacities are likely to be one of the reasons for the ecological success of this group of algae.

The concentration of CO_2_ in marine habitats is about 16 μM. This is lower than the K_M_ of the primary carboxylating enzyme, ribulose-1,5-bisphosphate carboxylase/oxygenase (Rubisco), that varies from 20 to 70 μM in diatoms^7^,^8^, and so may limit their photosynthesis. To circumvent this problem, diatoms have carbon dioxide concentrating mechanisms (CCMs) that elevate the CO_2_ concentration in the vicinity of the active site of Rubisco^8^. Different types of CCM are present in diatoms. In many species, the expression and/or activity of carbonic anhydrases (CAs) increase at low CO_2_ concentration^9^,^10^,^11^,^12^. *Phaeodactylum tricornutum*, a marine pennate diatom, has CO_2_/bicarbonate transporters^13^ and the genes encoding these transporters are also present in the genome of *Thalassiosira pseudonana,* a marine centric diatom^4^. In *Thalassiosira weissflogii*, there is evidence for a biochemical CCM, C_4_ metabolism but in other diatoms, different approaches, including ^14^C labeling^14^,^15^, protein localization^16^, transcriptomic^17^,^18^, enzyme measurements^12^ and proteomics^18^ have produced contradictory results.

Transcriptomic and proteomic approaches are powerful tools to understand the strategies used by diatoms that allow them to thrive in different environments, and have been used to study a number of species^19^,^20^. Among marine diatoms, *T. pseudonana*, and *P. tricornutum* are good models for these studies since their genome has been sequenced and annotated^4^,^21^. The response of *T. pseudonana* to nitrogen starvation was analysed using proteomics and shown to be different to that of the higher plant, *Arabidopsis thaliana*, and the green alga, *Chlamydomonas reinhardtii*^22^. In contrast, the increase of Krebs cycle enzymes under nitrogen starvation was similar in the diatom and in the cyanobacterium, *Prochlorococcus marinus*^22^. Proteomic and transcriptomic approaches have also been applied to study the effect of iron^23^,^24^, phosphorus^19^,^25^, light fluctuation^26^, silicate starvation^27^, oxidative stress^28^ and multiple stresses^29^.

Transcriptomic and proteomic studies that analyzed responses of diatoms to changes in CO_2_ concentration are relatively sparse even though inorganic carbon is a major resource for them. For example, when *P. tricornutum* was grown at elevated concentrations of bicarbonate, genes encoding carbonic anhydrase and bicarbonate transporter were up-regulated and lipid synthesis genes were down-regulated^30^. Proteomic analysis of *T. pseudonana* grown at different CO_2_ concentrations were used to propose a biochemical model for C4 photosynthesis and also showed that at low CO_2_, CA and other proteins involved in the CCM increased^18^.

The aim of this study was to understand more broadly how the proteome and the metabolism of *T. pseudonana* respond to low CO_2_ concentration. In order to compare the proteome of *T. pseudonana* exposed to different concentrations of CO_2_, one-dimensional gel electrophoresis (SDS-PAGE) and two-dimensional differential gel electrophoresis (2D-DiGE) followed by mass spectrometry were performed. In parallel, transcriptional changes and enzyme activities were analysed.

## Results

### Detection, identification and quantification of a protein expressed at low CO_2_

**Figure 1:**
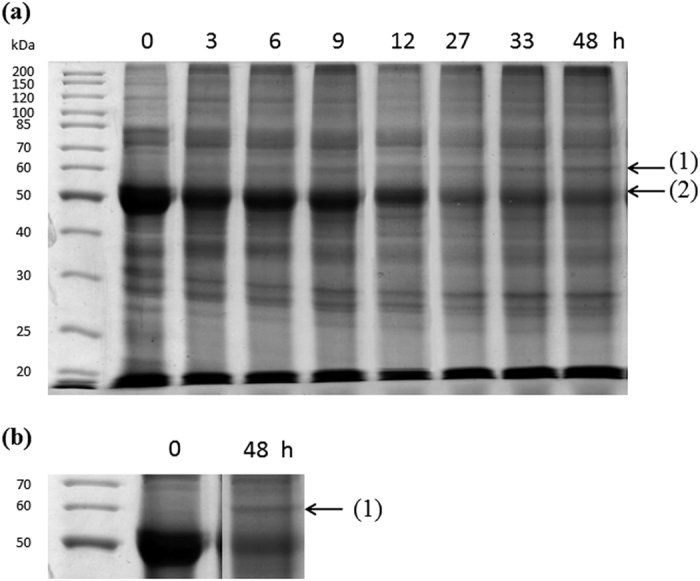
Changes in proteins from cells of *T. pseudonana* shifted from 20,000 to 50 ppm CO_2_ over 48 hours. (**a**) Proteins extracted at different times after switching the cells from 20,000 to 50 ppm CO_2_ were run on 12% SDS-PAGE and stained with Coomassie Blue. The first lane corresponds to molecular mass markers (Euromedex), and the other lanes correspond to proteins (20 μg) extracted at the times indicated. The arrows (1) and (2) indicate the position of the LCIP63 and Rubisco large subunit, respectively. (**b**) Close-up of the gel at the molecular mass of LCIP63 for the control and after 48 h treatment at low CO_2_.

Proteins in the crude extract obtained at different times after switching cells from high to low CO_2_ were analysed by one dimensional SDS-PAGE. There were no striking changes in protein expression, apart from a decrease of the large subunit of Rubisco (arrow 2, Fig. 1) and an increase of a protein with a molecular mass of about 60 kDa (arrow 1, Fig. 1) that was detected approximately 6 h after the switch (Fig. 1). This protein was also present at 400 ppm (data not shown).

**Figure 2:**
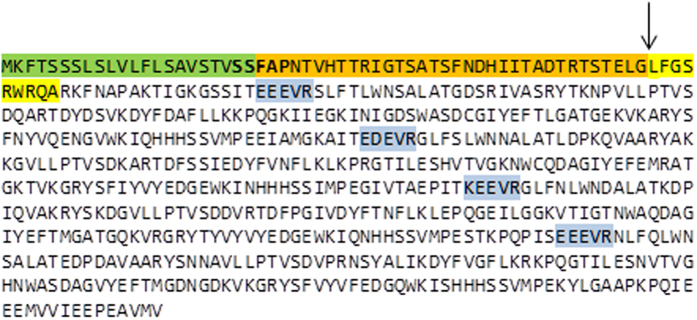
Sequence of LCIP63 identified in the genome of *T. pseudonana*. The 68.7 kDa protein sequence contains an endoplasmic reticulum signal peptide of 22 amino acid residues (in green), a chloroplast signal peptide of 34 amino acid residues (in orange) and the mature protein, the first nine amino acid residues of which were identified using N-terminal sequencing (in yellow), the arrow indicates the start of the mature protein. The sequence contained four COG4875 domains (see text), the start of which is highlighted in blue.

The band at about 60 kDa induced at low CO_2_, and also the slice of gel at the same position from the extract at high CO_2_, but without a band (control), were excised and analysed using mass spectrometry. Using *GENSCAN* software (http://genes.mit.edu/GENSCAN.html), a sequence corresponding to the protein induced by low CO_2_ with a molecular mass of 68.7 kDa was recovered (Fig. 2). This protein was identified with a high sequence coverage (58%) and a very high score (Protein Lynx Global Server (PLGS):16,400). The difference between the molecular mass estimated from the gel, of about 60 kDa, and the theoretical molecular mass of 68.7 kDa estimated from the genome, resulted from the presence of an N-terminal signal peptide (of about 6.0 kDa) cleaved in the mature protein. N-terminal sequencing identified the first nine amino acid residues from the mature protein, starting at LFGSR (Fig. 2, yellow), resulting in a theoretical molecular mass of 62.7 kDa. Therefore the protein is referred to as a Low CO_2_ Inducible Protein of 63 kDa hereafter called LCIP63. This confirmed that the 68.7 kDa protein sequence contains an N-terminal endoplasmic reticulum peptide signal of 22 amino acid residues (Fig. 2, green) and a chloroplast transit peptide of 34 amino acid residues (Fig. 2, orange; confirmed using Hectar software) and consistent with the transit peptide sequences for diatoms found before^31^,^32^. Sequence analysis of LCIP63 revealed four COG4875 related domains (uncharacterized protein conserved in bacteria with a cystatin-like fold) which are similar to Calcium/calmodulin dependent protein kinase II Association Domain (CaMKII_AD; Supporting data, Fig. S1). This protein sequence was also found in other marine diatoms, for example, *Thalassiosira oceanica* (four domains), *Pseudo-nitzschia multiseries* (three domains) and *P. tricornutum* (two domains) (Supporting data, Figs S2 to S4).

**Figure 3:**
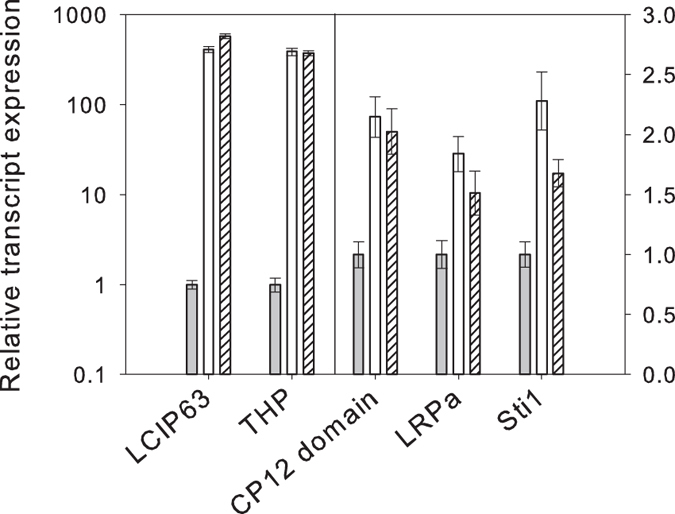
Relative transcript expression for proteins from cells of *T. pseudonana* at different CO_2_ concentrations. RNA was extracted from cells grown at 20,000 ppm (grey bar), switched to CO_2_ at 50 ppm for 12 h (white bar) and 24 h (hatched bar). Transcript levels are shown for LCIP63, (corresponding to spots 408 and 172, see Table 1), transmembrane hypothetical protein (THP, spot 696 and 422), “CP12 domain” protein (spot 926), light repressed protein a (LRPT, spot 378) and stress-inducible protein sti1 (spot 549). Error bars represent ± 1 SD.

RNA extraction followed by qPCR confirmed that the mRNA of LCIP63 was strongly up-regulated under low CO_2_ (Fig. 3) in contrast to the housekeeping gene for actin that did not change. In order to check if the response was related to carbon availability, rather than pH, cells were switched from high to low CO_2_ concentration while maintaining pH between 7 and 7.5 with 10 mM HEPES buffer. Transcript levels of LCIP63 were again highly upregulated (data not shown), suggesting that the response is directly triggered by low CO_2_.

### Analysis by 2D-DiGE of differentially expressed proteins upon a shift to low CO_2_

**Figure 4:**
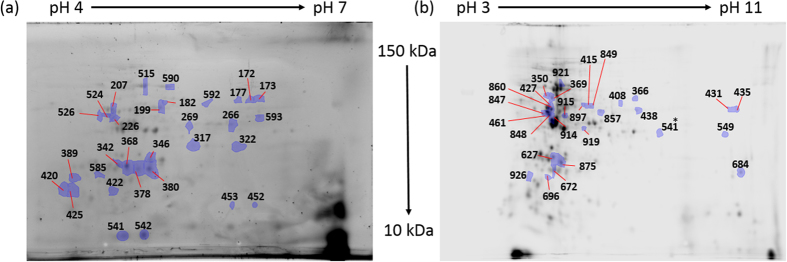
Differential expression of soluble proteins from *T. pseudonana* cells at 20,000 ppm and after switching to 50 ppm CO_2_ for 12 h. The gels were performed on two gradients of pH 4–7 (**a**) and pH 3–11 (**b**). Identification of the spots are given in Table 1.

**Figure 5:**
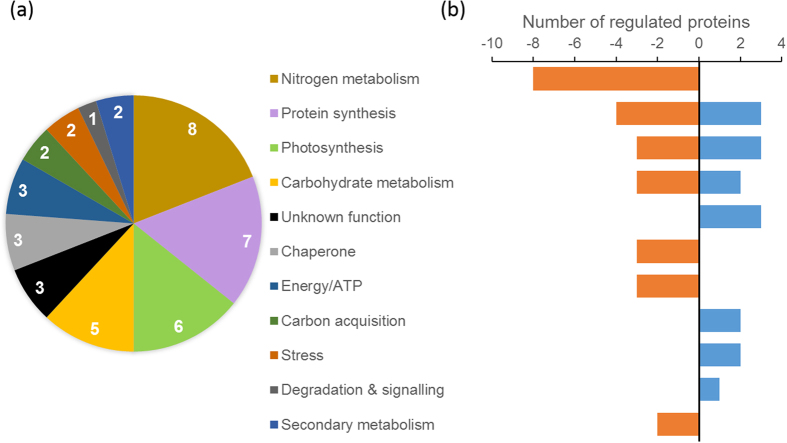
Protein regulation in *T. pseudonana* for different functional categories on a switch from 20,000 to 50 ppm CO_2_ for 12 hours. (**a**) Number of differentially expressed proteins by functional category, (**b**) number of proteins classified by functional category that increased (blue) or decreased (orange).

To check whether other proteins were differentially expressed in response to changing CO_2_ concentration, a more sensitive method than 1D SDS PAGE, 2D-DiGE, was performed using two different gradients (pH 4 to 7 and 3 to 11). Four biological replicates were combined and analysed, before, and 12 hours after, the switch to low CO_2_. There were statistically significant changes in abundance in low vs high CO_2_ concentration for 28 spots in each gradient (Fig. 4 and Table 1). These 56 spots were excised and analysed using mass spectrometry after trypsin digestion. The spots corresponded to 42 proteins, of which 16 were more abundant and 26 were less abundant, after the switch to low CO_2_. These proteins were categorized into 11 groups based on their biological function according to information from NCBI and UniProt (Fig. 5).

One δ carbonic anhydrase increased after the switch to low CO_2_ (Table 1). Another protein (Transmembrane Hypothetical Protein, THP) also increased after the switch to low CO_2_. It was identified using BLAST as a possible carbonic anhydrase though it did not bear the active site of this enzyme but only the transmembrane sequence. The mRNA expression for this protein also increased (Fig. 3).

Six proteins were found that are involved in photosynthesis (Table 1). Three of these are involved in the Calvin-Benson cycle and decreased on transfer to low CO_2_: the small subunit of Rubisco (in agreement with the pattern of the large subunit observed by SDS-PAGE), a predicted protein identified by BLAST as the chloroplast phosphoglycerate kinase (PGK) and the chloroplast glyceraldehyde-3-phosphate dehydrogenase, GAPDH_NADPH_. In contrast, ferredoxin-NADP reductase, the enzyme that catalyzes the last step of the primary phase of photosynthesis and produces NADPH, was more abundant after transfer to low CO_2_ (Table 1). Besides these enzymes, two proteins were also more abundant, a protein that possesses a CP12 domain and the mRNA expression of which also increased (Fig. 3) and a fucoxanthin-chlorophyll a/c light harvesting protein (Table 1).

Two proteins that are believed to be involved in stress responses were more abundant after transfer to low CO_2_: an ascorbate peroxidase (APX) and a predicted protein that could be a stress inducible protein named sti1 (Table 1). The expression of the mRNA encoding for the sti1 protein was checked and was also shown to be up-regulated (Fig. 3).

Three proteins that are involved in energy/ATP metabolism were less abundant after transfer to low CO_2_ (Table 1). These comprised two ATP synthases (one localized in the mitochondrion) and a predicted protein (regulation linked with ATP synthase). Three proteins classified in the carbohydrate metabolism group were less abundant after the transfer to low CO_2_: phosphofructokinase (PFK) from glycolysis, transketolase from the oxidative pentose phosphate pathway and an oxidoreductase. Meanwhile triose phosphate isomerase, also belonging to the carbohydrate metabolism group, increased.

Three chaperones (heat shock protein 70, 60 kDa chaperonin and mitochondrial chaperonin), as well as two proteins involved in secondary metabolism, were less abundant after transfer to low CO_2_ (Table 1). Two proteins involved in degradation and signaling increased after transfer to low CO_2_.

Four proteins involved in protein synthesis decreased while three others increased after the transfer to low CO_2_ (Table 1). Eight proteins involved in nitrogen metabolism including glutamine synthase, phosphoglycerate dehydrogenase and S-adenosyl methionine synthase, were less abundant after the transfer to low CO_2_. Three proteins that increased after the transfer to low CO_2_ were classified as proteins with unknown function, including LCIP63, found previously in the SDS-PAGE experiment (Fig. 1).

### Enzyme activity

**Figure 6:**
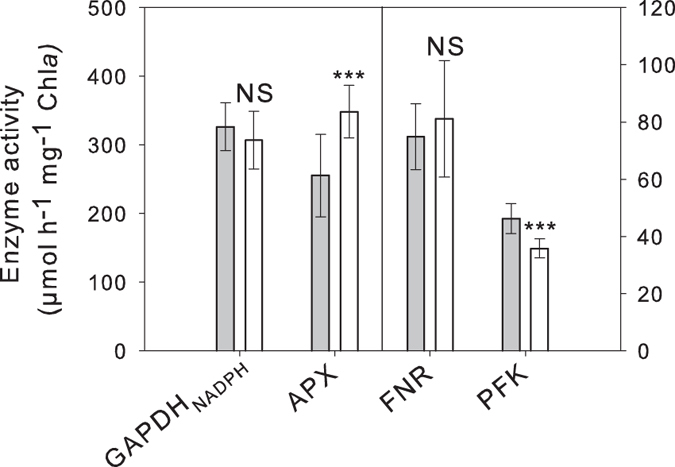
Enzyme activities from *T. pseudonana* at 20,000 and 50 ppm CO_2_. Activities of NADPH-dependent glyceraldehyde-3-phosphate dehydrogenase (GAPDH_NADPH_), ascorbate peroxidase (APX), ferredoxin NADP reductase (FNR) and phosphofructokinase (PFK) from cells grown at 20,000 ppm CO_2_ (grey bar), and after a shift to 50 ppm CO_2_ for 12 h (white bar). Error bars represent ± 1 SD. NS, not significant; ***P < 0.001.

To complement the 2D-DiGE results, the activity of four enzymes was measured on proteins extracted from cells grown at 20,000 ppm and 12 h after a switch to 50 ppm CO_2_ (Fig. 6). The changes in activity were consistent with the expression pattern observed using 2D-DiGE for APX and PFK since their activity increased by a factor of 1.36 (Student’s t-test, P < 0.001) and decreased by 1.29-fold (Student’s t-test, P < 0.001) respectively. However, FNR and GAPDH_NADPH_ activities were not statistically affected by the switch while in the 2D-DiGE experiments, the expression of FNR increased by 2.06-fold and that of GAPDH decreased by 1.88-fold.

**Figure 7:**
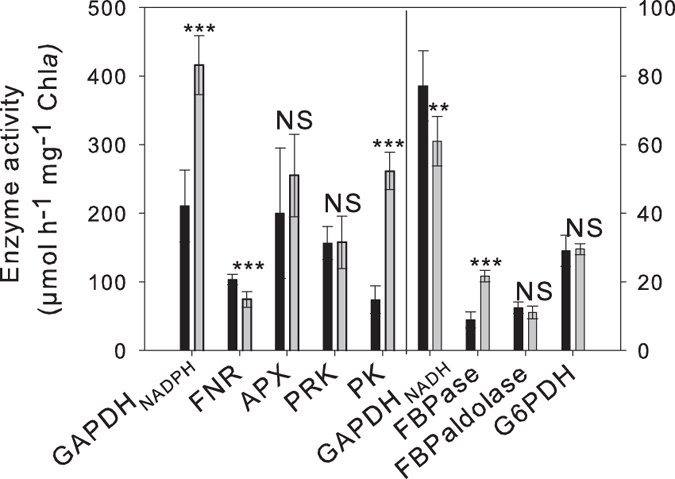
Enzyme activities from *T. pseudonana* grown at 20,000 and 400 ppm CO_2_. Activities of chloroplastic NADPH-dependent glyceraldehyde-3-phosphate dehydrogenase (GAPDH_NADPH_), ferredoxin NADP reductase (FNR), ascorbate peroxidase (APX), phosphoribulokinase (PRK), pyruvate kinase (PK), NADH-dependent glyceraldehyde-3-phosphate dehydrogenase (GAPDH_NADH_), fructose-1,6-bisphosphatase (FBPase), FBP aldolase and glucose-6-phosphate dehydrogenase (G6PDH) from cells grown in 400 ppm CO_2_ (black bars) and 20,000 ppm CO_2_ (grey bars). Error bars represent + 1 SD. **P < 0.01; ***P < 0.001.

Enzyme activities were also measured from cells acclimated to steady-state conditions at 20,000 and 400 ppm CO_2_ (Fig. 7). The activity of GAPDH_NADPH_ from the Calvin-Benson cycle was 1.97-fold lower at 400 ppm than at 20,000 ppm CO_2_ (Student’s t-test, P < 0.001) while the activity of FNR was 1.15-fold higher at 400 ppm than at 20,000 ppm CO_2_ (Student’s t-test, P < 0.001). At 400 ppm, the activity of fructose-1,6-bisphosphatase (FBPase) was 2.42-fold lower (Student’s t-test, P < 0.001) than at 20,000 ppm and the activity of pyruvate kinase (PK), a glycolytic enzyme that provides pyruvate to the Krebs cycle, was also lower (3.52-fold, Student’s t-test, P < 0.001). In contrast, the glycolytic GAPDH_NADH_, was 1.26-fold higher (Student’s t-test P < 0.01, P = 0.0016) at 400 than at 20,000 ppm. There were no significant differences in the activity of the other enzymes: phosphoribulokinase (PRK), fructose-1,6-bisphosphate (FBP) aldolase, APX, and glucose-6-phosphate dehydrogenase (G6PDH).

## Discussion

**Figure 8:**
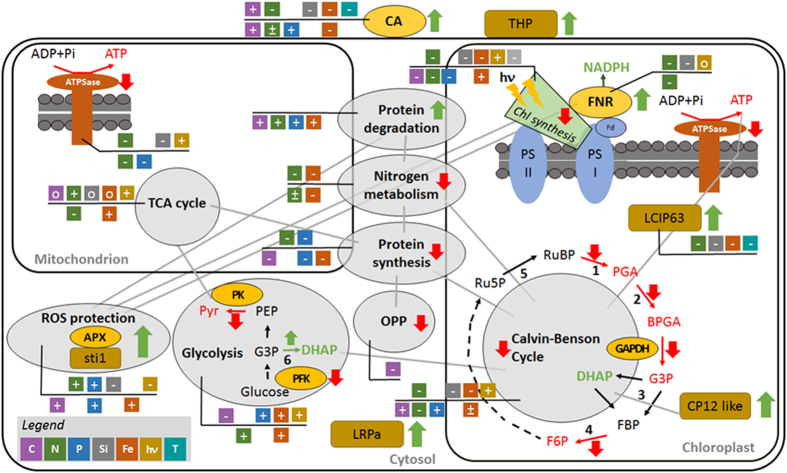
Schematic diagramme summarising the changes in *T. pseudonana* metabolism in response to limitation by inorganic carbon and other resources. Pathways are shown by grey circles and the component enzymes are shown as: 1, ribulose-1,5-bisphosphate carboxylase-oxygenase; 2, phosphoglycerate kinase; 3, fructose-bisphosphate aldolase; 4, fructose-1,6-bisphosphatase; 5, phosphoribulokinase; 6, triose-phosphate isomerase. The abbreviations used are: ADP, Adenosine diphosphate; APX ascorbate peroxidase; ATP, adenosine triphosphate; ATPSase, ATP synthase; BPGA, 1,3-bisphosphoglyceric acid; CA, carbonic anhydrase; Chl, chlorophyll; DHAP, dihydroxyacetone phosphate; F6P, fructose-6-phosphate; FBP, fructose-1,6-bisphosphate; Fd, ferredoxin; FNR, ferredoxin-NADP reductase; GAPDH, glyceraldehyde-3-phosphate dehydrogenase; G3P, glyceraldehyde-3-phosphate; h*v*, light; LCIP63, low CO_2_ inducible protein 63 kDa; LRPa, light-repressed protein a; NADPH, nicotinamide adenine dinucleotide phosphate (reduced); OPP, oxidative pentose phosphate pathway; PEP, phosphoenol pyruvate; PFK, phosphofructokinase; PGA, 3-phosphoglyceric acid; Pi, inorganic phosphate; PK, pyruvate kinase; PSI, photosystem I; PSII, photosystem II; Pyr, pyruvate; Ru5P, ribulose-5-phosphate; RuBP, ribulose-1,5-bisphosphate; Sti1, stress-inducible protein sti1; THP, transmembrane hypothetical protein. Changes in protein level in response to CO_2_-limitation are shown as increases (green arrows) or decreases (red arrows). Major interactions between the pathways and organelles are shown by grey lines. Also shown, as coloured boxes adjacent to proteins or pathways, are changes reported in the literature using proteomic or transcriptomic approaches in response to starvation by inorganic carbon (lilac) for *T. pseudonana*^18^; nitrogen (green) for *T. pseudonana*^22^,^29^, for *P. tricornutum*^50^; phosphorus (blue) for *T. pseudonana*^25^, for *P. tricornutum*^19^; silica for *T. pseudonana* (grey)^29^; iron (russet) for *T. pseudonana*^29^, for *P. tricornutum*^46^,^48^,^49^; fluctuating light (yellow-brown) for *T. pseudonana*^26^; temperature (turquoise) for *T. pseudonana*^22^,^29^. Responses for *T. pseudonana* (above the line) and for *P. tricornutum* (below the line) are denoted as an increase (+), decrease (−), both decrease and increase (±), no change (o). If the response is not commented on in the paper or if it is not obvious from Supplementary Information, no information is presented.

A holistic analysis was developed here using a range of approaches, to understand how the metabolism of *T. pseudonana* changes on transfer from high to low CO_2_. These changes, as well as those found in the literature for other stresses, are summarized in Fig. 8. The induction of a CCM under low CO_2_ is a well-known phenomenon that has both costs and benefits^33^, and therefore is strongly and rapidly regulated by growth conditions^34^. CA is very commonly more abundant at low CO_2_ in diatoms^9^,^10^,^11^,^18^,^35^, including *T. pseudonana* (this study and others^9^,^10^,^12^,^18^), green algae such as *C. reinhardtii*^36^ and cyanobacteria^37^. In our study, no CO_2_/bicarbonate transporters were regulated, as previously observed^18^, indicating that they play a less important role than CA in *T. pseudonana* in contrast to *P. tricornutum*^13^, *C. reinhardtii*^38^ and cyanobacteria^37^. Confirming our previous study^12^ but in contrast to a previous report^18^, we did not observe any changes in the abundance of enzymes involved in C4 metabolism.

Calvin-Benson cycle enzymes were less abundant when *T. pseudonana* cells were switched from high to low CO_2_. The small and the large subunit of Rubisco were less abundant which is in agreement with the changes of Rubisco activity obtained previously^12^. It is also consistent with the decrease of the 60 kDa chaperonin, shown here, a protein involved in Rubisco assembly, and with the lower Rubisco transcripts in *P. tricornutum* at 5 *vs* 15 mM bicarbonate^30^. In contrast, regulation of Rubisco is different in cyanobacteria. Rubisco was more abundant in *Synechocystis* PCC 6803 in air CO_2_ than in 3% CO_2_^37^ while no regulation was found when *Microcystis aeruginosa* cells were switched from 200 to 1,450 ppm^39^. These results indicate that diatoms may have developed strategies to cope with low CO_2_ that differ from cyanobacteria. This may be a consequence of the cyanobacterial CCM that involves polyhedral bodies, carboxysomes, that encapsulate Rubisco within a selectively permeable protein shell and simultaneously a CA for CO_2_ supply from a cytoplasmic bicarbonate pool^40^.

Other enzymes involved in the Calvin-Benson cycle were also differentially expressed. The observed decrease of NADPH-dependent GAPDH (GAPDH_NADPH_) at the protein level after the switch to low CO_2_, and the decrease in its activity at 400 ppm vs 20,000 ppm, are in good agreement with the lower GAPDH transcripts in *P. tricornutum* at 5 *vs* 15 mM bicarbonate^30^ and with GAPDH_NADPH_ activity changes in the freshwater diatom *A. formosa*^41^. Similarly, the chloroplastic PGK was less abundant after the CO_2_ switch to 50 ppm. PGK is involved in glycolysis and in the Calvin-Benson cycle, but the presence of the ASAF sequence^31^,^32^ at the start of the identified protein indicates that it was the chloroplastic isoform of PGK that responded. This result has not been found previously but is consistent with the down-regulation of other Calvin-Benson enzymes such as FBPase^18^. A protein, with a CP12 domain was more abundant and its transcript level was higher at low vs high CO_2_. Though CP12 proteins^42^ are widely studied for their role in photosynthesis, acting as assemblers for the GAPDH/PRK/CP12 complex^43^,^44^ and inhibiting these two Calvin-Benson enzymes within the supramolecular complex, they also play many other roles in plants, green algae and very likely in diatoms^45^,^46^. The increase in the protein with the CP12 domain may therefore represent an additional way of down-regulating the Calvin-Benson cycle, helping to match its activity to the CO_2_ supply (Fig. 8).

The tricarboxylic acid cycle, chrysolaminarin and lipid synthesis were not affected by short-term carbon limitation as was observed under nitrogen limitation^22^. In contrast, the glycolytic pathway was affected by the switch to low CO_2_. There was a decrease in activity of pyruvate kinase, a key enzyme of this pathway, and in the activity and abundance of PFK in agreement with Kutska *et al*.^18^. In contrast, triose phosphate isomerase expression increased at low CO_2_ which has been found before in plants under water stress^47^. Glycolytic metabolism increased in response to all other stresses in both *T. pseudonana* and *P. tricornutum* (Fig. 8). As has been found before^18^, an enzyme involved in the oxidative pentose phosphate pathway, transketolase, was less abundant which may reduce the availability of ribose, an intermediate required for nucleic acid synthesis. This is consistent with the decrease in protein synthesis (Fig. 8).

Changing the CO_2_ supply to *T. pseudonana* has a large effect on metabolic pathways other than those related to carbon. Indeed, the switch to low CO_2_ had a negative impact on proteins involved in nitrogen metabolism as in *T. pseudonana* and *P. tricornutum* exposed to iron and nitrogen starvation (Fig. 8 29,48,49,50). The decrease of proteins involved in nitrogen metabolism under low CO_2_ may be related to the homeostasis between carbon and nitrogen as shown in the higher plant, *Arabidopsis thaliana*^51^ and in diatoms^22^.

Overall, there is a decrease in many metabolic pathways under carbon limitation and this is also evidenced by the decrease in ATP synthase in both mitochondria and chloroplasts. This may result in a mismatch between energy production and the metabolic demand that in turn, may increase the risk of oxidative damage and reactive oxygen species (ROS) generation. To limit the damage, a number of changes are triggered. For instance, geranylgeranyl reductase, that catalyzes the most critical step in chlorophyll synthesis, was less abundant at low CO_2_ which may reduce ROS production by reducing light harvesting and electron pressure. Ferredoxin-NADP^+^ reductase (FNR), that produces NADPH in the last step of the primary phase of photosynthesis, was more abundant after the switch to 50 ppm CO_2_. This could increase the supply of NADPH, a reducing agent involved in protecting cells against ROS. Lastly, an increased abundance of ROS scavenging enzymes is a common response to oxidative stress. Indeed, the activity and abundance of APX increased after the switch to low CO_2._ The abundance and transcript level of another stress-responsive protein, sti1 was also higher as also observed under salt stress^52^. Moreover, as a consequence of carbon limitation, there was an increase of proteins involved in degradation and signaling, such as 14–3–3 proteins, multitasking proteins involved in the response of plants to many environmental stresses^53^,^54^. An increase in protein degradation has also been observed in *P. tricornutum* under different stresses (Fig. 8).

A protein found here, named LCIP63, increased substantially at the protein and transcript level when cells were grown at 400 ppm or switched to 50 ppm CO_2_. This protein was reported in a previous work as a “CaMKII associated domain containing protein” (SI Table 6^29^) but was not discussed. We analysed the sequence of this protein and found four COG4875 domain repetitions, similar to Ca^2+^/calmodulin activated protein kinase II (CaMKII)_association domain (AD). This domain is a C-terminal region of approximately 140 residues found in CaMKII that is responsible for holoenzyme oligomerization^55^. CaMKIIs are calmodulin-dependent protein kinases that are able, via their calmodulin (CaM) binding domain, to respond to calcium ions (Ca^2+^). Calcium ions are secondary messengers involved in many signalling pathways and in the response of cells to changing conditions in plants, macroalgae and diatoms^49^,^56^. Calmodulin is present in the chloroplasts of higher plants^57^, where it plays a role in the regulation of photosynthesis in response to light^58^, and CaMKIIs are present in green algae^59^ and in the dinoflagellate *Symbiodinium*^60^ that is, like diatoms, a member of the chromalveolates. Although the function of LCIP63 is presently unknown, the presence of a chloroplast signal peptide strongly suggests that it is located in the chloroplast because signal peptides in diatoms are very well defined^31^,^32^. Moreover in response to silicate, iron, nitrogen and temperature stress, this protein (Joint Genome Institute protein ID 264181) was less abundant, among the most affected proteins (up to 9-fold, SI Table 6^29^) and did not change under low CO_2_ probably because it was already present in the control treatment that was 400 ppm^29^. These different patterns suggest that LCIP63 is not involved in responses to general stress but instead is specifically present when CO_2_ is limited. The sequence of LCIP63 was also found in the genome of *T. oceanica, P. multiseries* and *P. tricornutum,* suggesting that it might play a widespread role in the responses of diatoms to low CO_2_.

To conclude, the response of diatoms to an individual stress requires many metabolic pathways to be altered in a co-ordinated way. In this study on carbon limitation (Fig. 8), these responses act to: (i) increase acquisition mechanisms for the limiting resource (e.g. CA increase), (ii) match the expense of the metabolic pathways to the requirement (e.g. a reduction in the Calvin-Benson cycle and protein synthesis), (iii) redirect resources (e.g. protein degradation to recycle nitrogen and carbon), (iv) minimise damage caused by excess light energy (e.g. by reducing light acquisition and stimulating ROS scavengers), and (v) co-regulate the metabolic pathways (e.g. homeostasis between carbon and nitrogen). These responses appear to be common to different stresses such as nutrient limitation (nitrogen, phosphorus, silica, iron) or temperature decrease and fluctuating light (Fig. 8). Under those stresses, there is a tendency, in *T. pseudonana*, for protein, chlorophyll and ATP synthesis to be down-regulated, protein degradation, glycolysis and ROS protection to be up-regulated and for the tricarboxylic acid cycle to be unaffected (Fig. 8). Furthermore, where a comparison can be made, the responses of *T. pseudonana* and *P. tricornutum* are broadly similar. Mechanisms are required to choreograph these changes. LCIP63, described here, could take this role for carbon stress since it contains a domain for a signaling protein, Calcium/calmodulin dependent protein kinase II, and responded similarly to carbonic anhydrase, which is a marker for carbon-limitation. The role of LCIP63 in carbon-limitation is further supported by results in SI Table 6^29^ since it was less abundant under nitrogen, phosphorus and iron limitation and low temperature: conditions that minimise carbon limitation. However, the precise mode of action of this protein remains to be elucidated.

While up to now work has mainly been focussed on individual stresses in the real world diatoms are likely to experience multiple stresses. The co-ordinated response of many metabolic pathways to a single stress, shown here and elsewhere, strongly indicates that in the future diatoms should be challenged with multiple stresses in order to obtain a clearer picture of how they respond to their changing environment.

## Methods

### Strain, media and culture conditions

*Thalassiosira pseudonana*, (CCAP 1085/12; equivalent to CCMP1335), was grown in artificial sea water and F/2+ Si medium at 16 °C under continuous illumination at 50 μmol photon m^−2^ s^−1^, photosynthetically active radiation, as described earlier^12^. Cultures were grown at steady-state CO_2_ concentrations of 20,000 or 400 ppm. In ’switch’ experiments, cells grown at 20,000 ppm were transferred to 50 ppm for different lengths of time. Cultures were bubbled at a gas flow rate of 130 mL min^−1^.

### Protein extraction and identification by mass spectrometry of LCIP63

Soluble protein extracts were prepared as described previously^5^,^61^ in a buffer containing 30 mM Tris, 4 mM EDTA (called TA buffer), 1 mM NAD, 20% glycerol and 40 μg mL^−1^ protease inhibitor (Sigma). The protein concentration was assayed using the Bio-Rad (Hercules, CA, USA) reagent using bovine serum albumin as a standard.

Extracts were incubated for 15 min at 80 °C with 10% sodium dodecyl sulfate (SDS), 10 mM DTT, 20% glycerol, 0.2 M Tris and 0.05% bromothymol blue. Protein migration was performed on 12% polyacrylamide gel Mini-PROTEAN^®^ Tetra Cell (Biorad, Hercules, USA). Gels were either stained with Coomassie blue to determine any differential pattern at low and high CO_2_ or the proteins were transferred onto polyvinylidene fluoride (PVDF) membranes, stained with red Ponceau, and used for N-terminal sequencing. LCIP63 was identified using a Synapt G1 mass spectrometer (Waters, Manchester, UK) coupled to a nano flow UPLC nanoAcquity (Waters). Spectra and protein search were processed by the PLGS 3.0.1 software (Waters) with the same search parameters as described below. N-terminal sequence determination was performed by Edman degradation using an automatic sequencer (Procise 494, Applied Biosystems). Mature sequence of LCIP63 was analysed using Hectar (http://webtools.sb-roscoff.fr/) to identify the location of the protein.

### RNA quantification

*T. pseudonana* cells were centrifuged at 3,720 g for 10 min at 4 °C, frozen with liquid nitrogen and stored at −80 °C. 100 mg of cells, were incubated with 4 mL of Trizol^®^ reagent (ThermoFisher scientific, Waltham, USA), for 5 min and 800 μL of chloroform (Carlo Erba, Milan, Italy) was added. The mixture was shaken for 10 min at room temperature and centrifuged at 16,000 g for 15 min at 4 °C. To 600 μL of the aqueous phase, 375 μl of RNA dilution buffer (SV total RNA isolation system, Promega, Madison, USA) was added, mixed and centrifuged at 16,000 g for 10 min. 250 μL of 90% cold ethanol was added to the supernatant and next step performed from step 7 onwards following manufacturer’s SV total RNA isolation system kit protocol (Promega, Madison, USA). Isolated RNA was quantified spectrophotometrically at 260 nm (NanoDrop 2000c; Thermo Fisher Scientific) and stored at −80 °C. For cDNA synthesis, 175 ng total RNA and 0.5 μg random primers (Promega) were used with the GoScript™ Reverse transcriptase (Promega) according to the manufacturer’s instruction. Quantitative real-time PCR (qPCR) analyses were performed on a CFX96 Real-Time System (Bio-Rad, USA). The reaction volume was 15 μL and the final concentration of each primer was 0.5 μM. The cycling parameters of the qPCR were 98 °C for 2 min, followed by 45 cycles of 98 °C for 5 s, 60 °C for 10 s. A final melting curve from 65 °C to 95 °C was performed to determine the specificity of the amplification. To determine the amplification kinetics of each product, the fluorescence derived from the incorporation of EvaGreen into the double-stranded PCR products was measured at the end of each cycle using the SsoFast EvaGreen Supermix 2X Kit (Bio-Rad, USA). The results were analysed using Bio-Rad CFX Manager software, version 3.0 (Bio-Rad, USA). The RNA actin gene was used as a normalization reference. For each point, a technical duplicate and biological triplicate was performed. The amplification efficiencies for each primer pairs were between 75 and 100%. All of the primer pairs used for qPCR are reported in Table S1.

### 2D-DiGE and mass spectrometry analysis

All samples (150 μg) were washed with the 2D-Clean-up kit (GE Healthcare, Little Chalfont, UK) and solubilized in lysis buffer (8 M urea, 2 M thiourea, 4% (w/v) CHAPS, pH 8.5 without DTT and carrier ampholytes) to a final concentration of 2.5 μg μL^−1^. 50 μg of protein were labeled using 400 pmol of the dye following GE Healthcare protocol. The samples were vortexed and incubated for 10 min in the dark. Labeled samples were then combined, and 65 μM DTT (final concentration) was added. The samples were vortexed and kept for 10 min on ice in the dark. The combined samples were supplemented with an equal volume of double strength lysis buffer with IPG Buffer pH 4–7 or pH 3–11 (GE Healthcare) and then supplemented with Destreak Rehydration Buffer containing 0.5% (v/v) IPG buffer pH 4–7 to a final volume of 200 μL.

Isoelectric focusing (IEF) was performed on a mixture of 150 μg of protein per gel (50 μg for each treatment plus 50 μg of standard) using 11 cm gels with an immobilized linear pH gradient of 4–7 or 3–11 (Immobiline DryStrips, GE Healthcare) on an IPGphorIII machine (GE Healthcare). IEF was performed at a 300 V gradient for 1.5 h, a 1,000 V gradient for 1.5 h, a 6,000 V gradient for 2.5 h and a 6,000 V step for 2 h at 20 °C. Prior to SDS PAGE, IPG strips were equilibrated for 10 min in 6 M urea, 50 mM Tris pH 8.8, 2% SDS, 38.5% glycerol and 65 mM DTT, followed by 10 min with 2% iodoacetamide instead of DTT. The second dimension was performed using a Criterion Dodeca Cell separation unit (Biorad) and precast criterion TGX at 17 °C. IPG strips were placed on the top of the precast gels, overlaid with 0.5% agarose in 2x TGS running buffer (Biorad) containing bromophenol blue. Gels were run at 20 °C using TGS 1x running buffer. Electrophoresis was conducted at 50 V for 5 h. After SDS-PAGE, cyanine dye-labeled protein gels were scanned directly using the EDI scanner (GE Healthcare) at a resolution of 50 μm. After fluorescence imaging, gels were washed for 10 min in ultrapure water and post-stained with the ImperialTM Protein Stain (Thermo Scientific, Rockford, USA). The Samespot software package (version 4.0.3; nonlinear dynamics) was used to determine protein abundance and statistics based on 2D-DiGE. Based on Samespot analysis, spots of interest were excised using a Shimadzu Biotech Xcise System (Champs sur Marne, France) and submitted to trypsin digestion after reduction and alkylation. Tryptic peptides were analysed on a Q-Exactive Plus mass spectrometer (ThermoFisher) coupled to a nano liquid chromatography (Ultimate 3000, Dionex). Briefly, peptides were separated by a 30 min-linear gradient of 80% acetonitrile in 0.1% formic acid in water, on a reversed phase C18 column, and were detected on the mass spectrometer in a positive ion mode, alternating a scan event full MS in the Orbitrap analyser at 70 000 resolution in a 350–1900 m/z range with scan events of MS/MS (Top 10), in the Higher Energy Collisional Dissociation cell at 17 500 resolution in a 200–2000 m/z range. For mass spectra processing and protein searches, Proteome Discoverer (version 2.1.0.81, ThermoFisher) was used and the raw files generated from the MS/MS analyses were searched with Sequest HT and Mascot search engines. Search parameters were: *T. pseudonana* extracted from the protein database NCBI (ID 35128, 24,090 entries); Enzyme trypsin; maximum two miscleavages;carbamidomethylation of cysteine and oxidation of methionine set as static and dynamic modifications, respectively; precursor and fragment mass tolerance set at ± 5–10 ppm and ± 0.02 Da, respectively. Proteins were identified if 2 unique “rank 1” peptide sequences of more than 6 amino acids passed the high confidence filter, with validation on q-Value (Strict Target FDR: 0.01) and maximum Delta Cn: 0.05 (Sequest HT criteria), or the peptide individual score was above 20 and p < 0.05 (Mascot criteria).

### Enzyme activities measurement

Enzyme activities were measured using extracts from exponentially growing cells. The soluble protein extracts were prepared as described in Erales *et al*.^61^ and Mekhalfi *et al*.^5^ in the TA buffer containing 0.1 mM NAD and 1 mM cysteine. Activities were measured from the rates of appearance or disappearance of 0.2 mM NADH or NADPH at 340 nm at room temperature (20 to 25 °C). All biochemicals were obtained from Sigma Inc (Saint Louis, MO, USA). GAPDH_NADPH_ and GAPDH_NADH_ were measured as described in Graciet *et al*.^62^. Soluble protein extracts were also incubated with 20 mM dithiothreitol (DTT) and/or 1 mM NADPH for 10 min. PRK was measured as described previously^63^. PFK, G6PDH, and Ferredoxin NADP reductase (FNR) was measured as described in Mekhalfi *et al*.^5^. PK was measured in the presence of 5 mM PEP, LDH (2 U) and 1 mM ADP and 0.2 mM NADH. FBP aldolase was measured as described in Erales *et al*.^64^. Ascorbate peroxidase (AP) was measured in 0.2 M Tris/HCl buffer pH 7.8, in the presence of 0.25 mM ascorbic acid and 0.5 mM H_2_O_2_. Activity was monitored at 290 nm using an extinction coefficient of 2.8 mM^−1^ cm^−1^.

## Additional Information

**How to cite this article**: Clement, R. *et al*. Responses of the marine diatom *Thalassiosira pseudonana* to changes in CO_2_ concentration: a proteomic approach. *Sci. Rep.* **7**, 42333; doi: 10.1038/srep42333 (2017).

**Publisher's note:** Springer Nature remains neutral with regard to jurisdictional claims in published maps and institutional affiliations.

## Supplementary Material

Supplemental Information

## Figures and Tables

**Table 1:** Changes in protein abundance and transcript levels on selected genes after *T. pseudonana* cells were switched from 20,000 to 50 ppm CO_2_.

Accession No. (NCBI)	Accession No. (Swiss Prot)	Gel	Spot ID	Annotation	Functional group	Protein fold change	RNA fold change
220971674	B8C995	3–11	435	Translation elongation factor alpha	Protein synthesis	4.11	−
220973545	B8C239	3–11	431	RL4e, ribosomal protein 4e 60S large ribosomal subunit	Protein synthesis	3.86	−
220976737	B8BTB5	4–7	422	Transmembrane hypothetical protein (THP)	Carbon acquisition	3.71	388
220970433*	B8CCH0*	4–7	172	LCIP63	Unknown function	3.60	409
220969212	B8CGE1	3–11	549	Stress-inducible protein sti1 (BLAST)	Stress	3.42	2.27
220976737	B8BTB5	3–11	696	Transmembrane hypothetical protein (THP)	Carbon acquisition	3.41	388
209583601	B5YLS7	4–7	368	Triose-phosphate isomerase	Carbohydrate metabolism	3.20	−
220978199	B8BRE6	4–7	378	Light-repressed protein a	Protein synthesis	2.90	1.83
220969257	B8CFA9	4–7	380	Ascorbate peroxidase	Stress	2.61	−
220970433*	B8CCH0*	3–11	408	LCIP63	Unknown function	2.40	409
220972037	B8C7S8	4–7	585	Hypothetical protein THAPSDRAFT_7881	Unknown function	2.32	−
220978087	B8BQS1	3–11	926	Protein with CP12 domain (BLAST)	Photosynthesis	2.29	2.15
220978087	B8BQS1	4–7	425	Protein with CP12 domain (BLAST)	Photosynthesis	2.23	2.15
220975793	B8BX06	3–11	627	14–3–3-like protein	Degradation & signalling	2.13	−
220969044	B8CGL9	4–7	317	Ferredoxin-NADP reductase	Photosynthesis	2.06	−
220974564	B8BZK1	4–7	342	Phosphomannomutase	Carbohydrate metabolism	2.06	−
220976367	B8BU33	3–11	875	Fucoxanthin-chlorophyll a/c light-harvesting protein	Photosynthesis	1.99	−
220969044	B8CGL9	3–11	541*	Ferredoxin-NADP reductase	Photosynthesis	1.74	−
220969727	B8CEN5	4–7	389	Glycoprotein fp21 (BLAST)	Unknown function	1.67	−
589908182	W8VYH0	4–7	420	Delta carbonic anhydrase	Carbon acquisition	1.65	−
220974490	B8BZ41	4–7	266	Phosphofructokinase	Carbohydrate metabolism	−1.45	−
224011563	B5YLQ5	3–11	366	Mitochondrial chaperonin	Chaperone	−1.51	−
125987721	A0T0R6	4–7	207	ATP synthase CF1 beta chain	Energy/ATP	−1.54	−
209586260	B5YN92	3–11	914	Phosphoglycerate kinase (BLAST)	Photosynthesis	−1.55	−
220968997	B8CGK1	4–7	588	Glutamine synthetase (BLAST)	Nitrogen metabolism	−1.56	−
220970599	B8CCE1	4–7	322	Pseudouridylate synthase	Protein synthesis	−1.57	−
125987749	A0T0 × 9	3–11	684	30S ribosomal protein S3, chloroplastic	Protein synthesis	−1.58	−
209586260	B5YN92	3–11	915	Phosphoglycerate kinase (BLAST)	Photosynthesis	−1.60	−
220976316	B8BTR4	4–7	515	Transketolase	Carbohydrate metabolism	−1.61	−
220969226	B8CGI1	3–11	672	Demethylmenaquinone methyltransferase (BLAST)	Secondary metabolism	−1.61	−
220968997	B8CGK1	4–7	590	Glutamine synthetase (BLAST)	Nitrogen metabolism	−1.61	−
220968642	B8LCI4	4–7	593	Phosphoglycerate dehydrogenase (BLAST)	Nitrogen metabolism	−1.63	−
220975991	B8BY55	4–7	199	S-adenosylmethionine synthetase	Nitrogen metabolism	−1.65	−
220970584	B8CCA0	4–7	269	Aspartate-ammonia ligase	Nitrogen metabolism	−1.65	−
209586260	B5YN92	3–11	461	Phosphoglycerate kinase (BLAST)	Photosynthesis	−1.68	−
220973284	B8C635	3–11	369	Heat shock protein 70	Chaperone	−1.69	−
220969617	B8CET1	3–11	921	PEFG, plastid translation factor EF-G (BLAST)	Protein synthesis	−1.70	−
118411188	A0T0 × 0	3–11	427	60 kDa chaperonin	Chaperone	−1.71	−
125987721	A0T0R6	3–11	860	ATP synthase CF1 beta chain	Energy/ATP	−1.76	−
220977307	B8BT02	4–7	233	Aspartate aminotransferase	Nitrogen metabolism	−1.77	−
220970665	B8CCS6	3–11	438	Serine hydroxymethyltransferase	Nitrogen metabolism	−1.80	−
118411103	A0T0N5	4–7	541	Rubisco small subunit	Photosynthesis	−1.82	−
209583569	B5YLM0	4–7	182	Histidinol dehydrogenase	Nitrogen metabolism	−1.82	−
220977520	B8BQU2	3–11	919	Glyceraldehyde-3-phosphate dehydrogenase precursor	Photosynthesis	−1.88	−
220970292	B8CDB3	3–11	857	Geranylgeranyl reductase (BLAST)	Secondary metabolism	−1.92	−
220972213	B8C6C6	3–11	849	Mitochondrial ATPase, inner membrane	Energy/ATP	−1.98	−
118411103	A0T0N5	4–7	542	Rubisco small subunit	Photosynthesis	−2.00	−
220968222	B8LDL7	4–7	592	N-acetyl-glutamate-gamma-semialdehyde dehydrogenase	Nitrogen metabolism	−2.07	−
220971973	B8C8K9	4–7	526	RS1, ribosomal protein 1, partial	Protein synthesis	−2.07	−
209585841	B5YMV8	3–11	350	Regulation, link with ATP synthase (BLAST)	Energy/ATP	−2.19	−
125987721	A0T0R6	3–11	415	ATP synthase CF1 beta chain	Energy/ATP	−2.22	−
220977926	B8BT70	3–11	847	Oxidoreductase	Carbohydrate metabolism	−2.28	−
220977307	B8BT02	4–7	524	Aspartate aminotransferase	Nitrogen metabolism	−2.29	−
220977307	B8BT02	4–7	226	Aspartate aminotransferase	Nitrogen metabolism	−2.56	−
209586260	B5YN92	3–11	848	Phosphoglycerate kinase (BLAST)	Photosynthesis	−2.66	−
220972213	B8C6C6	3–11	897	Mitochondrial ATPase, inner membrane	Energy/ATP	−3.43	−

The proteins highlighted in this study were selected stringently by combining different criteria: the highest confident peptide spectral matches obtained by mass spectrometry, and by matching experimental isoelectric points, and molecular masses to theoretical values.
