# Supplementary material for: Responses of the marine diatom Thalassiosira pseudonana to changes in CO2 concentration: a proteomic approach
Source: Sci Rep. 2017 Feb 9;7:42333. doi: 10.1038/srep42333 (PMC5299434; doi:10.1038/srep42333)
Supplement: Supplemental Information [file srep42333-s1.pdf]

Supplemental Data from the original manuscript by Clement *et al*. entitled

Responses of the marine diatom *Thalassiosira pseudonana* to changes in CO_2 concentration: a proteomic approach

Romain Clement[1], Sabrina Lignon[2], Pascal Mansuelle[2], Erik Jensen[1], Mathieu Pophillat[3], Regine Lebrun[2], Yann Denis[4], Carine Puppo[1], Stephen C. Maberly[5] & Brigitte Gontero[1,*]

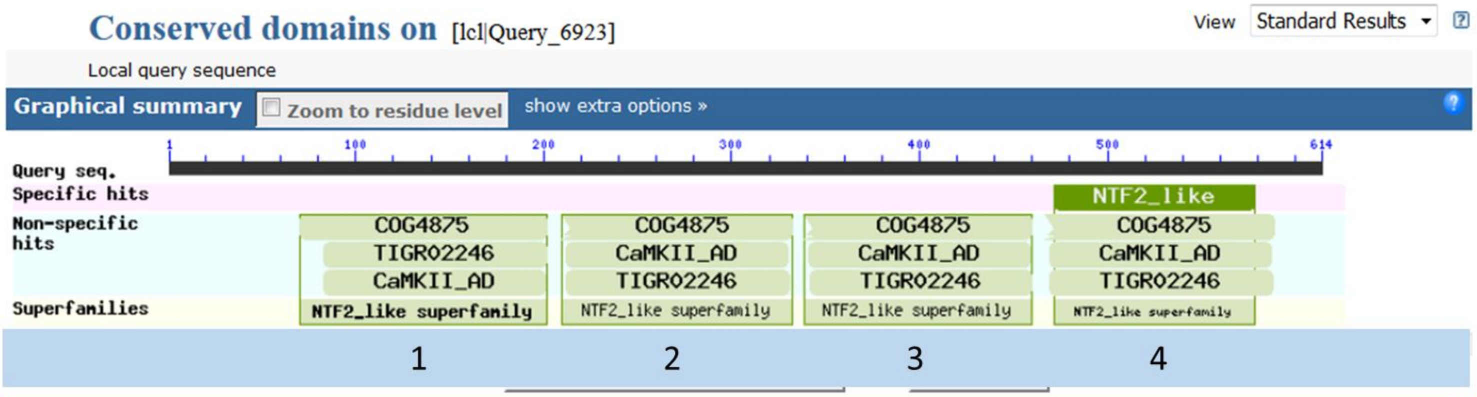

List of domain hits

	Name	Accession	Description	Interval	E-value
4	[+] COG4875	COG4875	Uncharacterized protein [Function unknown];	467-589	8.45e-43
3	[+] COG4875	COG4875	Uncharacterized protein [Function unknown];	339-460	2.35e-40
2	[+] COG4875	COG4875	Uncharacterized protein [Function unknown];	210-332	3.22e-40
1	[+] COG4875	COG4875	Uncharacterized protein [Function unknown];	70-202	1.73e-35
4	[+] CaMKII_AD	pfam08332	Calcium/calmodulin dependent protein kinase II association domain; This domain is found at the ...	469-587	2.87e-18
4	[+] TIGR02246	TIGR02246	conserved hypothetical protein; This family consists of uncharacterized proteins found in a ...	469-588	6.94e-18
2	[+] CaMKII_AD	pfam08332	Calcium/calmodulin dependent protein kinase II association domain; This domain is found at the ...	212-330	1.80e-14
1	[+] TIGR02246	TIGR02246	conserved hypothetical protein; This family consists of uncharacterized proteins found in a ...	83-202	2.25e-14
3	[+] CaMKII_AD	pfam08332	Calcium/calmodulin dependent protein kinase II association domain; This domain is found at the ...	341-459	2.50e-14
3	[+] TIGR02246	TIGR02246	conserved hypothetical protein; This family consists of uncharacterized proteins found in a ...	342-460	2.01e-13
1	[+] CaMKII_AD	pfam08332	Calcium/calmodulin dependent protein kinase II association domain; This domain is found at the ...	83-201	2.44e-13
2	[+] TIGR02246	TIGR02246	conserved hypothetical protein; This family consists of uncharacterized proteins found in a ...	212-331	2.10e-12
4	[+] NTF2_like	cd00531	Nuclear transport factor 2 (NTF2-like) superfamily. This family includes members of the NTF2 ...	472-578	2.64e-04

Figure S1. Conserved domains within LCIP63 sequence from *T. pseudonana*. Domains present in the LCIP 63 were identified using BLAST. LCIP63 contains four domains. On the left side of the list are mentioned numbers (1,2,3 and 4) that correspond to each domain. The E values allow to discriminate between the different possible domains (COG4875, Calcium/calmodulin dependent protein kinase II association domain, the lower E-value, corresponding to the most significant score and alignment.

```
>tr|B7G7G0|B7G7G0_PHATC Predicted protein OS=Phaeodactylum tricornutum
(strain CCAP 1055/1) GN=PHATRDRAFT_39236 PE=4 SV=1
MKIGYLANVLVAISLSSASGFELRRFSATSTFPGGQLTYVTPFTKVVKQKDTTQLRMGIR
SFIRGKKLTVNHSNDNKNLTGPTKNEAEIRALFSLWNNALATGDSRLVAARYASDAVLLP
TVSDIPRTNYDLIKNYFDAFLLKKPQGVIKEGYIKLGKGWAQDNGIYEFTMGIDNTKVKA
RYSFVYTKENGKWKIAHHHSSMMPEGITVGRPIEEEEVKNLFHLWNDALATGDSRVVADR
YAKTSVLLPTVSDVPRTDYDGIKDYFDAFLLKKPQGVILESNVVIGANWAQDAGLYEFTM
GVDGSKVQARYTFVYVFEDGQWKISHHHSSVMPEAFLGPAAPANANTEVVSKK
```

```
CLUSTAL O(1.2.1) multiple sequence alignment


LCIP63          MKFTSSSLSLVLFLSAVSTVSSFAPNTVHTT--RIG---TSATSFNDHIITADTRTSTEL
tr|B7G7G0       MKIG--YLANVLVAISLSSASGFELRRFSATSTFPGGQLTYVTPFTKVVK-QKDTTQLRM
                **:    *: **.  ::*:.*.*  . . :*    *   * .* *.. :   .  *. .:

LCIP63          GLFGSRWRQARKFN---APAKTIGKGSSITEEEVRSLFTLWNSALATGDSRIVASRYTKN
tr|B7G7G0       GIR--SFIRGKKLTVNHSNDNKNLTGPTKNEAEIRALFSLWNNALATGDSRLVAARYASD
                *:    : :.:*:.   :  :.  .* : .* *:*:**:***.********:**:**:.:

LCIP63          PVLLPTVSDQARTDYDSVKDYFDAFLLKKPQGKIIEGKINIGDSWASDCGIYEFTLGATG
tr|B7G7G0       AVLLPTVSDIPRTNYDLIKNYFDAFLLKKPQGVIKEGYIKLGKGWAQDNGIYEFTMGIDN
                 ********  **:** :*:************ * ** *::*..**.* ******:*

LCIP63          EKVKARYSFNYVQENGVWKIQHHHSSVMPEEIAMGKAITEDEVRGLFSLWNNALATLDPK
tr|B7G7G0       TKVKARYSFVYTKENGKWKIAHHHSSMMPEGITVGRPIEEEEVKNLFHLWNDALATGDSR
                 ******** *.:*** *** *****:*** *::*: * *:**: ** ***:**** * :

LCIP63          QVAARYAKKGVLLPTVSDKARTDFSSIEDYFVNFLKLKPRGTILESHVTVGKNWCQDAGI
tr|B7G7G0       VVADRYAKTSVLLPTVSDVPRTDYDGIKDYFDAFLLKKPQGVILESNVVIGANWAQDAGL
                 ** ****..********  ***:..*:***  **  **:*.****.*.:* **.****:

LCIP63          YEFEMRATGKTVKGRYSFIYVYEDGEWKINHHHSSIMPEGIVTAEPITKEEVRGLFNLWN
tr|B7G7G0       YEFTMGVDGSKVQARYTFVYVFEDGQWKISHHHSSVMPEAFLGPAAPANANTEVVSKK--
                *** * . *..*:.**:*:**:***:***.*****:***.::     :: :.. : :

LCIP63          DALATKDPIQVAKRYSKDGVLLPTVSDDVRTDFPGIVDYFTNFLKLEPQGEILGGKVTIG
tr|B7G7G0       ------------------------------------------------------------


LCIP63          TNWAQDAGIYEFTMGATGQKVRGRYTYVYVYEDGEWKIQNHHSSVMPESTKPQPISEEEV
tr|B7G7G0       ------------------------------------------------------------


LCIP63          RNLFQLWNSALATEDPDAVAARYSNNAVLLPTVSDVPRNSYALIKDYFVGFLKRKPQGTI
tr|B7G7G0       ------------------------------------------------------------


LCIP63          LESNVTVGHNWASDAGVYEFTMGDNGDKVKGRYSFVYVFEDGQWKISHHHSSVMPEKYLG
tr|B7G7G0       ------------------------------------------------------------


LCIP63          AAPKPQIEEEMVVIEEPEAVMV
tr|B7G7G0       ----------------------
```

Figure S2. Similarity of sequence found in *P. tricornutum* and LCIP63 sequence. Alignment of protein (B7G7G0, UniProt database ID) with LCIP63 was performed using MUSCLE software (http://www.ebi.ac.uk/Tools/msa/muscle/). Only two domains are present on this protein as shown by the alignment.

```
>tr|K0SPX5|K0SPX5_THAOC Uncharacterized protein OS=Thalassiosira oceanica
GN=THAOC_16311 PE=4 SV=1
MGRWPRRGGAGVDVPRLLARATTLMSRFLASTRIFRSRAAAHSPSPNNSNMKFSTVAVAL
STLSSTQGFSPVAPNRGRSMAQRGVVNVEPSSTELNLFGSKRLFNSPAKVDGDITEKEVK
ALFELWNQALATGDSRIVASRYTKSPVLLPTVSDVPRTDFASVKDYFDSFLLKQPQGKII
ESHVNIGEGWASDVGIYEFTMGATGDKVKGRYSYNYVKEDGIWKIQHHHSSVMPEEIAMG
KPITDDEVRGLFSLWNNALATLDPKQVANRYAKTGVLLPTVSDTPRADYGAIEDYFVNFL
KLKPQGEILESYVTVGHNWCQDVGIYEFAMGATGKKVKGRYSFIYVYEDGEWKIQHHHSS
IMPEGIVTAEPITEKEVRGLFNLWNDALATLDPKKVADRYSKEGVLLPTVSDIPRTDYPG
IEDYFTNFLKLKPQGEIESGNIIVGTNWAQDAGIYEFTMGATGAKVKGRYTFVYVFEDGE
WKISQHHSSVMPEASKPKEISEEEVKNLFQLWNSALATEDPDAVAKRYASKAVLLPTVSD
VPRTDYALIKDYFVGFLKKKPQGEILESNVTIGHNWCQDAGIYEFTMGATGDKVKGRYSF
VYVYEDGEWKISHHHSSVMPEAFLGPAPKPAVDAKETVSA
```

```
CLUSTAL O(1.2.1) multiple sequence alignment


LCIP63          --------------------------------------------------MKFTSSSLSL
tr|K0SPX5       MGRWPRRGGAGVDVPRLLARATTLMSRFLASTRIFRSRAAAHSPSPNNSNMKFSTVAVA-
                                                                  ***:: :::

LCIP63          VLFLSAVSTVSSFAPNTVHTTRIGTSATSFNDHIITADTRTSTELGLFGSRWRQARKFNA
tr|K0SPX5       ---LSTLSSTQGFSPVAPNRG------RSMAQRGVVNVEPSSTELNLFGSK----RLFNS
                   **::*:...*:* : .         *: :: :.    :**** ****:    * **:

LCIP63          PAKTIGKGSSITEEEVRSLFTLWNSALATGDSRIVASRYTKNPVLLPTVSDQARTDYDSV
tr|K0SPX5       PAKVD---GDITEKEVKALFELWNQALATGDSRIVASRYTKSPVLLPTVSDVPRTDFASV
                ***.    ..***:**::** ***.****************.*********  ***: **

LCIP63          KDYFDAFLLKKPQGKIIEGKINIGDSWASDCGIYEFTLGATGEKVKARYSFNYVQENGVW
tr|K0SPX5       KDYFDSFLLKQPQGKIIESHVNIGEGWASDVGIYEFTMGATGDKVKGRYSYNYVKEDGIW
                *****:****:*******.::***:.**** ******:****:***.***:***:*:*:*

LCIP63          KIQHHHSSVMPEEIAMGKAITEDEVRGLFSLWNNALATLDPKQVAARYAKKGVLLPTVSD
tr|K0SPX5       KIQHHHSSVMPEEIAMGKPITDDEVRGLFSLWNNALATLDPKQVANRYAKTGVLLPTVSD
                ****************** **:*********************** ****.*********

LCIP63          KARTDFSSIEDYFVNFLKLKPRGTILESHVTVGKNWCQDAGIYEFEMRATGKTVKGRYSF
tr|K0SPX5       TPRADYGAIEDYFVNFLKLKPQGEILESYVTVGHNWCQDVGIYEFAMGATGKKVKGRYSF
                . *:*:.:*************:* ****:****:*****.***** * ****.*******

LCIP63          IYVYEDGEWKINHHHSSIMPEGIVTAEPITKEEVRGLFNLWNDALATKDPIQVAKRYSKD
tr|K0SPX5       IYVYEDGEWKIQHHHSSIMPEGIVTAEPITEKEVRGLFNLWNDALATLDPKKVADRYSKE
                ***********:******************::*************** ** :**.****:

LCIP63          GVLLPTVSDDVRTDFPGIVDYFTNFLKLEPQGEILGGKVTIGTNWAQDAGIYEFTMGATG
tr|K0SPX5       GVLLPTVSDIPRTDYPGIEDYFTNFLKLKPQGEIESGNIIVGTNWAQDAGIYEFTMGATG
                *********  ***:*** *********:***** .*:: :*******************

LCIP63          QKVRGRYTYVYVYEDGEWKIQNHHSSVMPESTKPQPISEEEVRNLFQLWNSALATEDPDA
tr|K0SPX5       AKVKGRYTFVYVFEDGEWKISQHHSSVMPEASKPKEISEEEVKNLFQLWNSALATEDPDA
                 **:****:***:*******.:********::**: ******:*****************

LCIP63          VAARYSNNAVLLPTVSDVPRNSYALIKDYFVGFLKRKPQGTILESNVTVGHNWASDAGVY
tr|K0SPX5       VAKRYASKAVLLPTVSDVPRTDYALIKDYFVGFLKKKPQGEILESNVTIGHNWCQDAGIY
                ** **:.:************..*************:**** *******:****..***:*

LCIP63          EFTMGDNGDKVKGRYSFVYVFEDGQWKISHHHSSVMPEKYLGAAPKPQIEEEMVVIEEPE
tr|K0SPX5       EFTMGATGDKVKGRYSFVYVYEDGEWKISHHHSSVMPEAFLGPAPKPAVDAKETVSA---
                ***** .*************:***:************* :** **** :: : .*

LCIP63          AVMV
tr|K0SPX5       ----
```

Figure S3. Similarity between sequence found in *T.oceanica* and LCIP63 sequence. Alignment of protein (K0SPX5, UniProt database ID) with LCIP63 was performed using MUSCLE software (http://www.ebi.ac.uk/Tools/msa/muscle/). The four domains are present on this protein as shown by the alignment.

```
>jgi|Psemu1|261071|estExt_Genewise1Plus.C_5280010
MSFDFVPVRKKPVVRKVVVEKAPVKAPVKASAPAEAAPFFVDETRTANSAVDITEKEVRSLFSLWNNALA
TGDSRLVASRYATKSGAVLLPTVSDTPRTDYDSIKDYFDGFLKKQPQGEILDGNIRIGNGWAQDAGIYEF
TMGAEGGAKVKGRYTYTYVMEDGQWKIAHHHSSVMPEGIDIATSITKEEVQGLFHLWNDALATGDSALVA
SRYASKGVLLPTVSDVPRDDFDSIKDYFDAFLLKEPQGKILESFVTIGTNWAQDVGIYEFTMGATGAKVK
ARYSFVYTFEGGQWKIAHHHSSQMPEEVVPGPASITNEEVRGLFYLWNDALATLDPAQVASRYSVETAPC
LLPTVSDVPRTDYDSIMAYFVDFCKKEPQGEILESYVTVGHNWAMDDGIYEFTMGATGDKVKARYSFVYT
LEDGVWKIAHHHSSQMPEEIVPKSTVPEFAEANGSSSD*
```

```
CLUSTAL O(1.2.2) multiple sequence alignment


LCIP63              MKFTSSSLSLVLFLSAVSTVSSFAPNTVHTTRIGTSATSFNDHIITADTRTSTELGLFGS
Ps|jgi261071        MSF------------------DFVPVRKK--PVVRK-VVVEKAPVKAPVKAS-------A
                    *.*                  .*.*   :   :  . . .:.  :.* .::*       :

LCIP63              RWRQARKFNAPAKTIGKGSSITEEEVRSLFTLWNSALATGDSRIVASRYTK--NPVLLPT
Ps|jgi261071        PAEAAPFFVDETRTANSAVDITEKEVRSLFSLWNNALATGDSRLVASRYATKSGAVLLPT
                      . *  *   ::*  .. .***:******:***.********:*****:.    *****

LCIP63              VSDQARTDYDSVKDYFDAFLLKKPQGKIIEGKINIGDSWASDCGIYEFTLGATG-EKVKA
Ps|jgi261071        VSDTPRTDYDSIKDYFDGFLKKQPQGEILDGNIRIGNGWAQDAGIYEFTMGAEGGAKVKG
                    ***  ******:*****.** *:***:*::*:*.**:.**.*.******:** *  ***.

LCIP63              RYSFNYVQENGVWKIQHHHSSVMPEEIAMGKAITEDEVRGLFSLWNNALATLDPKQVAAR
Ps|jgi261071        RYTYTYVMEDGQWKIAHHHSSVMPEGIDIATSITKEEVQGLFHLWNDALATGDSALVASR
                    **::.** *:* *** ********* * :..:**::**:*** ***:**** *   **:*

LCIP63              YAKKGVLLPTVSDKARTDFSSIEDYFVNFLKLKPRGTILESHVTVGKNWCQDAGIYEFEM
Ps|jgi261071        YASKGVLLPTVSDVPRDDFDSIKDYFDAFLLKEPQGKILESFVTIGTNWAQDVGIYEFTM
                    **.**********  * **.**:***  **  :*:*.****.**:*.**.**.***** *

LCIP63              RATGKTVKGRYSFIYVYEDGEWKINHHHSSIMPEGIVTA-EPITKEEVRGLFNLWNDALA
Ps|jgi261071        GATGAKVKARYSFVYTFEGGQWKIAHHHSSQMPEEVVPGPASITNEEVRGLFYLWNDALA
                     *** .**.****:*.:* *:*** ***** *** :* .   **:******* *******

LCIP63              TKDPIQVAKRYSKD--GVLLPTVSDDVRTDFPGIVDYFTNFLKLEPQGEILGGKVTIGTN
Ps|jgi261071        TLDPAQVASRYSVETAPCLLPTVSDVPRTDYDSIMAYFVDFCKKEPQGEILESYVTVGHN
                    * ** ***.*** :    *******  ***: .*: **.:* * ******* . **:* *

LCIP63              WAQDAGIYEFTMGATGQKVRGRYTYVYVYEDGEWKIQNHHSSVMPESTKPQPISEEEVRN
Ps|jgi261071        WAMDDGIYEFTMGATGDKVKARYSFVYTLEDGVWKIAHHHSSQMPEEIVPKSTVPEFAEA
                    ** * ***********:**:.**::**. *** *** .**** ***.  *:    * ..

LCIP63              LFQLWNSALATEDPDAVAARYSNNAVLLPTVSDVPRNSYALIKDYFVGFLKRKPQGTILE
Ps|jgi261071        -----NGSS--SD-----------------------------------------------
                         *.:   .*

LCIP63              SNVTVGHNWASDAGVYEFTMGDNGDKVKGRYSFVYVFEDGQWKISHHHSSVMPEKYLGAA
Ps|jgi261071        ------------------------------------------------------------


LCIP63              PKPQIEEEMVVIEEPEAVMV
Ps|jgi261071        --------------------
```

Figure S4. Similarity between sequence found in *Pseudo-nitzschia multiseries* CLN-47 and LCIP63 sequence. Alignment of protein (261071, JGI database ID) with LCIP63 was performed using MUSCLE software (http://www.ebi.ac.uk/Tools/msa/muscle/). Only three domains are present on this protein as shown by the alignment.

Table S1. List of primers used for qRT PCR.

Gene ID name, forward and reverse primers
Actin (ACT1) : Gene ID 7449411
CCTCGATTCGGGAGATGGTG
ATCAGGGAGCTCGAAGGACT
LCIP63 :
CCCTCGATCCCAAGCAAGTT
CTCCAAGATGGTTCCTCGGG
THP : Gene ID 7452158
ATGCTAACGCTCTCGCTACC
ACCTCCAAGTCAGCACACAG
CP12 protein : Gene ID 7445028
TGTTGATATCCGTGCACCCC
TATCCCTCAGCCACGATTGC
LRPa : Gene ID 7446790
CAAGGAGGCCATTTTCGCAC
CTGAATGAGGCCGACTCCAC
Sti1 : Gene ID 7448899
TAGGTTGAACAGGACGGTGC
CACCAACCATTGCACTCTGC
